# Mechanisms of regulatory T cell infiltration in tumors: implications for innovative immune precision therapies

**DOI:** 10.1136/jitc-2021-002591

**Published:** 2021-07-29

**Authors:** Shohei Koyama, Hiroyoshi Nishikawa

**Affiliations:** 1 Division of Cancer Immunology, Research Institute/Exploratory Oncology Research and Clinical Trial Center (EPOC), National Cancer Center, Tokyo/Chiba, Japan; 2 Department of Respiratory Medicine and Clinical Immunology, Osaka University Graduate School of Medicine, Osaka, Japan; 3 Department of Immunology, Nagoya University Graduate School of Medicine, Nagoya, Japan

**Keywords:** immunotherapy, tumor microenvironment, CD4-positive T-lymphocytes, costimulatory and inhibitory molecules

## Abstract

With the broad application of cancer immunotherapies such as immune checkpoint inhibitors in multiple cancer types, the immunological landscape in the tumor microenvironment (TME) has become enormously important for determining the optimal cancer treatment. Tumors can be immunologically divided into two categories: inflamed and non-inflamed based on the extent of immune cell infiltration and their activation status. In general, immunotherapies are preferable for the inflamed tumors than for non-inflamed tumors. Regulatory T cells (Tregs), an immunosuppressive subset of CD4^+^ T cells, play an essential role in maintaining self-tolerance and immunological homeostasis. In tumor immunity, Tregs compromise immune surveillance against cancer in healthy individuals and impair the antitumor immune response in tumor-bearing hosts. Tregs, therefore, accelerate immune evasion by tumor cells, leading to tumor development and progression in various types of cancer. Therefore, Tregs are considered to be a crucial therapeutic target for cancer immunotherapy. Abundant Tregs are observed in the TME in many types of cancer, both in inflamed and non-inflamed tumors. Diverse mechanisms of Treg accumulation, activation, and survival in the TME have been uncovered for different tumor types, indicating the importance of understanding the mechanism of Treg infiltration in each patient when selecting the optimal Treg-targeted therapy. Here, we review recent advances in the understanding of mechanisms leading to Treg abundance in the TME to optimize Treg-targeted therapy. Furthermore, in addition to the conventional strategies targeting cell surface molecules predominantly expressed by Tregs, reagents targeting molecules and signaling pathways specifically employed by Tregs for infiltration, activation, and survival in each tumor type are illustrated as novel Treg-targeted therapies. The effectiveness of immune precision therapy depends on conditions in the TME of each cancer patient.

## Introduction

The interaction between tumor cells and immune cells plays an important role in tumor development and progression. Tumor cells with low immunogenicity are selected by immunological pressure (immune selection) and immunosuppressive molecules and cells are employed to hinder antitumor immunity (immune escape), which is the basic principle of cancer immunoediting.^1^ Cancer immunotherapies, such as immune checkpoint inhibitors that can resurge impaired antitumor immune responses during tumor development and progression, have been widely used in multiple cancer types clinically.^2^ However, their therapeutic efficacy is limited because of immune evasion mechanisms other than checkpoint molecules, such as immunosuppressive cells in the tumor microenvironment (TME) and disrupted antigen presentation of tumor-specific antigens, which originate from gene alterations in tumor cells. Gene alterations in tumor cells modulate cell-intrinsic signaling involved in tumor cell proliferation, secretion of immunomodulatory molecules for immune cell infiltration and function, and presentation of non-self tumor-specific antigens stemming from gene alterations shape the immunological landscape, leading to tumors with or without inflammation: inflamed versus non-inflamed of tumors. Inflamed tumors and non-inflamed tumors are also called hot and cold tumors, respectively. Non-inflamed tumors include the immune excluded type and the immune desert type. Immune cells are present at the invasive margins of the tumors in the immune excluded type. Immune cells are hardly detected throughout tumors in the immune desert type. Concisely, this review focuses on inflamed and non-inflamed tumors.

Regulatory T cells (Tregs) are a distinctive lineage of CD4^+^ T cells that suppress the immune system. They restrain immune responses against self-antigens in autoimmunity and excessive immune-mediated inflammation during infection.^3^ Tregs function as major immunosuppressive cells in the context of tumor immunity. They efficiently infiltrate and adapt to the TME and dampen antitumor immune responses. Tregs promote tumor cell proliferation through inhibiting antigen-presenting cells (APCs), consuming a critical cytokine for effector T cell activation and function, and producing immunosuppressive humoral factors, resulting in the development of an immunosuppressive TME.^4–7^ Therefore, while abundant cytotoxic CD8^+^ T cells (CTLs) among tumor-infiltrating lymphocytes (TILs) are generally correlated with favorable prognosis and clinical response to immunotherapies, a higher proportion of Tregs among TILs is associated with poor prognosis and clinical response to immunotherapies.^8–10^ An abundance of Tregs is frequently observed in the TME with multiple types of cancer, both in inflamed and non-inflamed tumors, yet, the mechanisms leading to Treg abundance remain to be elucidated. Recent studies revealed that multiple mechanisms are involved in Treg infiltration, activation, and survival in the TME, which varies by tumor type. Since the immunological characteristics of the TME is closely linked to gene alterations in tumor cells, understanding the mechanisms leading to Treg abundance in each patient becomes critical for developing Treg-targeted therapy. In this review, we illustrate recent findings regarding the mechanisms leading to Treg abundance in the TME and how Tregs suppress antitumor immune responses. We also discuss which Treg-targeting therapies could be optimal from the perspective of immune/genomic precision medicine.

## Characteristics and phenotypes of Tregs

Forkhead box P3 (FOXP3) is a master regulatory transcription factor for generating the immunosuppressive CD4^+^ Treg lineage, which maintains self-tolerance and immunological homeostasis.^11–14^ Tregs consist of two distinct subsets based on where they are generated. Thymic Tregs (tTregs), also called natural Tregs (nTregs), originate from the thymus. Peripheral Tregs (pTregs), also called induced Tregs (iTregs), are derived from naive CD4^+^ T cells in the periphery under certain conditions, such as T cell receptor (TCR) stimulation in the presence of cytokines such as tumor growth factor beta (TGF-β), interleukin (IL)-2, and retinoic acid.^15–18^ In contrast to tTregs, pTregs are unstable and convert to conventional FOXP3^−^ CD4^+^ T cells (Tconvs) with the loss of FOXP3 expression. DNA hypomethylation in the *Foxp3* conserved noncoding sequences 2 (CNS2) locus, where some transcription factors including signal transducer and activator of transcription 5 (STAT5) and cyclic AMP (cAMP) response element-binding protein interact and induce FOXP3 expression, is observed in tTregs but not in pTregs, which contributes to the stability of FOXP3 expression in tTregs.^19^ Although both tTregs and pTregs are found in tumors and restrain antitumor immune responses,^20 21^ details about the function and stability of each Treg subset in the TME remain to be elucidated.

**Figure 1:**
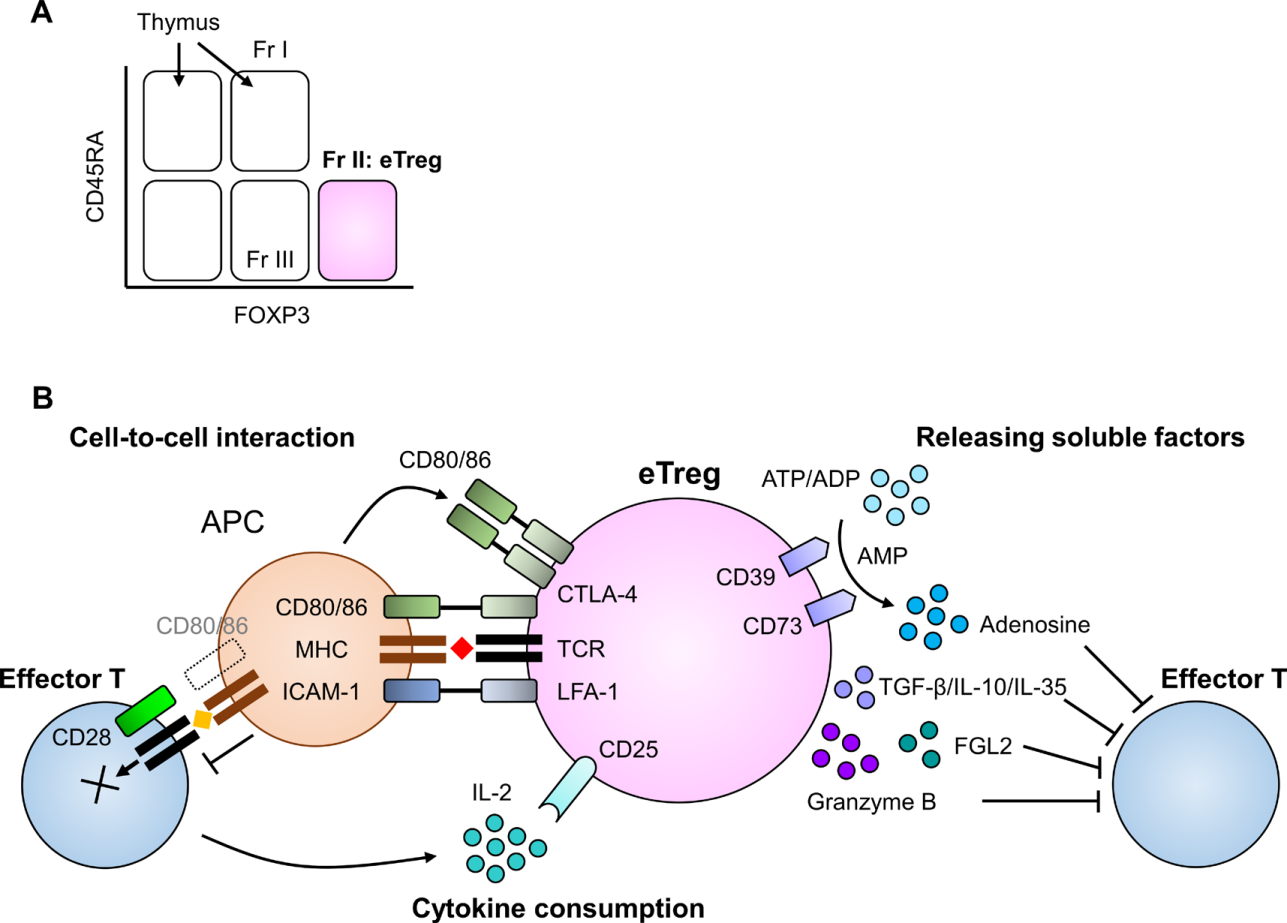
Mechanisms of immunosuppression by eTregs. (A) FOXP3^+^CD4^+^ T cells can be classified into three fractions based on FOXP3 (and/or CD25) and CD45RA expression levels: fraction 1 (Fr. 1), naive Tregs defined as FOXP3^low^(CD25^low^)CD45RA^+^ cells; fraction 2 (Fr. 2), eTregs, defined as FOXP3^high^(CD25^high^)CD45RA^−^ cells; and fraction 3 (Fr. 3), non-Tregs, defined as FOXP3^low^(CD25^low^)CD45RA^−^ cells. Naive Tregs (Fr. 1) that recently left the thymus have weak immunosuppressive activity. Once naive Tregs (Fr. 1) receive TCR stimulation, they differentiate into eTregs (Fr. 2), which have strong immunosuppressive activity. Non-Tregs (Fr. 3) do not have immunosuppressive activity. (B) Coinhibitory receptor cytotoxic T lymphocyte antigen 4 (CTLA-4) in Tregs inhibits costimulatory signaling via CD80/B7-1 and CD86/B7-2 in antigen presenting cells (APCs) due to its high affinity binding to CD80/B7-1 and CD86/B7-2. when these costimulatory molecules interact with CTLA-4, they are captured from APCs by transendocytosis. Compared with effector T cells, Tregs harbor receptors with higher affinity for IL-2: much higher CD25 expression in Tregs than in effector T cells. This provides Tregs with a competitive advantage in utilizing the limited amount of IL-2 in the TME. Tregs produce TGF-β, IL-10, and IL-35 for immunosuppression. TGF-β reduces the cytotoxic function of effector T cells. Fgl2 secreted by Tregs binds to FcγRIIB in CD8^+^ T cells and leads to their apoptosis. CD39 and CD73 expressed on the cell surface of Tregs act as ectonucleotidases that hydrolyze ATP or ADP to AMP and AMP to adenosine, respectively. Adenosine suppresses effector T cells. eTregs, effector Tregs; FcγRIIB, Fc fragment of IgG receptor IIb; FOXP3, Forkhead box P3; IL-10, interleukin 10; TCR, T cell receptor; TGF-β, tumor growth factor beta; TME, tumor microenvironment; Tregs, regulatory T cells.

While TCR stimulation in the presence of TGF-β easily induces FOXP3 expression in human naive T cells in vitro, unlike in mice, these FOXP3-expressing cells fail to gain immunosuppressive function. Instead, they produce proinflammatory cytokines on stimulation.^22^ Thus, we need to carefully understand how TGF-β-induced FOXP3^+^ T cells in mice and humans differ: In this review, we mainly describe human FOXP3^+^ T cells. Accordingly, FOXP3^+^CD4^+^ T cells in humans include populations that are heterogeneous in phenotype and function. Therefore, specific markers to discriminate Tregs from Tconvs are indispensable for evaluating human FOXP3^+^CD4^+^ T cells. A classification of human FOXP3^+^CD4^+^ T cells based on FOXP3 (and/or CD25) and CD45RA expression was proposed by Dr. Sakaguchi’s group: Fraction 1 (Fr. 1), naive Tregs defined as FOXP3^low^(CD25^low^)CD45RA^+^ cells; Fraction 2 (Fr. 2), effector Tregs (eTregs), defined as FOXP3^high^(CD25^high^)CD45RA^−^ cells; and Fraction 3 (Fr. 3), non-Tregs, defined as FOXP3^low^(CD25^low^)CD45RA^−^ cells^23^ (figure 1A). Naive Tregs (Fr. 1) that recently left the thymus have weak immunosuppressive activity. Once naive Tregs (Fr. 1) receive TCR stimulation, they differentiate into eTregs (Fr. 2), which have strong immunosuppressive activity. It has been recently shown that Tregs in the TME harbor a unique TCR repertoire. Tregs and Tconvs, which recognize tumor-specific antigens,^21^ have different TCR repertoires. These findings suggest that the differentiation and expansion of eTregs in the TME are mainly induced by stimulation from immunogenic self-antigens specific for tTregs in tumor cells as Tregs recognize immunogenic self-antigens.^24 25^ Non-Tregs (Fr. 3) do not have immunosuppressive properties. Instead, they can produce inflammatory cytokines, such as interferon (IFN)-γ and IL-17.^23^ In the TME of melanoma, non-small cell lung cancer (NSCLC), and gastric cancer, eTregs are heavily infiltrated and account for 20%–60% of CD4 ^+^T cells^26^. tTregs can also be detected in the peripheral blood. The proportion of tTregs among CD4^+^ T cells declines with age: 4%–10% in cord blood, 1%–4% in young adults, and 0.5%–1.5% in healthy elderly individuals.^27^ By contrast, the proportion of eTregs increases with age: 0%–0.5% in cord blood, 1%–2.5% in young adults, and 1%–4% in elderly health individuals.^23^

## Mechanisms through which Tregs suppress antitumor immune responses

Tregs hinder antitumor immune responses through multiple mechanisms (figure 1B). In Tregs, coinhibitory receptor cytotoxic T lymphocyte antigen 4 (CTLA-4) disrupts costimulatory signaling via CD80/B7-1 and CD86/B7-2 in APCs and the co-stimulatory receptor CD28 in effector T cells through higher affinity binding of CTLA-4 to CD80/B7-1 and CD86/B7-2 than CD28 to CD80/B7-1 and CD86/B7-2. When these co-stimulatory molecules interact with CTLA-4, they are captured from APCs such as dendritic cells (DCs) via trans-endocytosis, resulting in impaired costimulation via CD28.^28^ In addition, leukocyte function-associated antigen 1 in Tregs forms long-lasting aggregates with DCs as a result of the disrupted interaction between DCs and effector T cells.^29^

IL-2 is an essential cytokine for the survival of both Tregs and effector T cells. Compared with effector T cells, Tregs can dominantly access IL-2 with a higher affinity receptor that consists of α (CD25), β (CD122), and γ (CD132) subunits.^30^ Although TCR and IL-2 signaling are necessary for the immunosuppressive activity of Tregs,^31 32^ Tregs themselves are not able to produce IL-2 because FOXP3 suppresses the transcription of IL-2.^33 34^ By contrast, FOXP3 induces the expression of CD25, a high-affinity receptor for IL-2.^33^ Tregs harbor much higher CD25 expression than effector T cells, which provides Tregs with a competitive advantage in utilizing the limited amount of IL-2 in the TME.^35^ As a result, there is more accumulation of Tregs than effector T cells in the TME.

In addition, secretion of immunosuppressive molecules including immunosuppressive cytokines from Tregs also suppresses antitumor immunity. Tregs produce TGF-β, IL-10, and IL-35 for immunosuppression. TGF-β reduces the cytotoxic function of NK cells and CTLs^36^ and induces the conversion of NK cells into type 1 innate lymphoid cells in the TME, which fail to control tumor growth and metastasis.^37^ TGF-β signaling also drives the trans-differentiation of Th17 cells into Tregs, resulting in the development of immune tolerance and immunosuppression in the TME.^38^ TGF-β can be produced by both immune and non-immune cells. The function of Treg cell-derived TGF-β remains controversial. While several studies have implicated TGF-β as playing an important role in immunosuppression by Tregs,^39–41^ other studies have shown that Treg-derived TGF-β1, the major subtype of TGF-β, is largely redundant in immune regulation.^42 43^ Further investigation is warranted to clarify the detailed role of Treg-derived TGF-β1 in vivo.

Tregs are a major source of the immunomodulatory cytokine IL-10 in the TME. Treg-specific ablation of IL-10 in a murine model exhibited tissue-specific inflammation in the colon, lung, and skin, but not in the systemic inflammatory phenotype. These findings indicate that Treg-derived IL-10 might be important for regulating inflammation at environmental interfaces.^44^ Although IL-10 exerts various effects in the TME, its immunosuppressive effect can be enhanced with IL-35, which is also produced by Tregs. In a murine melanoma model, IL-35-producing Tregs accumulated in the TME and disrupted antigen-specific effector T cell activation and their effector function via falling them into the exhaustion.^45^ The expression pattern and immunosuppressive roles of IL-10 and IL-35 in tumor-infiltrating Tregs were different in tumor-bearing mice and patients with NSCLC.^46^ Tumor growth was slower in mice with IL-35^-/-^IL-10^-/-^ Tregs compared with those with either IL-35^-/-^ or IL-10^-/-^ Tregs. Interestingly, IL-35 and IL-10 have different immunomodulatory functions. IL-35-producing Tregs promote the exhaustion of effector T cells whereas IL-10-producing Tregs inhibit the cytotoxic effector function of effector T cells.^45 46^

Fibrinogen-like protein 2 (FGL2) is produced by Tregs. It binds to the Fc fragment of IgG receptor IIb (FcγRIIB) receptor, which transduces inhibitory signaling via an immunoreceptor tyrosine-based inhibitory motif. Because FcγRIIB can be induced in activated CD8^+^ T cells in the TME, FGL2 secreted by Tregs binds to FcγRIIB in CD8^+^ T cells and leads to their apoptosis through caspase 3 and 7 induction. Mice with FcγRIIB-deficient CD8^+^ T cells have less tumor growth than control mice,^47^ suggesting that Tregs enable suppression of CD8^+^ T cells through FcγRIIB. Granzyme B produced by cytotoxic cells, such as NK cells and CTLs, is an important effector molecule for killing target cells. However, a subset of Tregs in the TME produce granzyme B and kill effector cytotoxic cells, which can also be involved in Treg-mediated suppression of antitumor immune responses.^40^

CD39 and CD73 expressed on the cell surface of Tregs act as ectonucleotidases that hydrolyze ATP or ADP to AMP and AMP to adenosine, respectively.^48^ ATP hydrolysis by CD39 and CD73 produces adenosine and suppresses effector T cells.^49 50^ Although ATP is strictly retained within cells under normal conditions, intracellular ATP can be released from necrotic or inflammatory cells in tumors via vesicular exocytosis and membrane transporters.^51^ There are two classes of P2 purinergic receptors for ATP: P2XR and P2YR. While both are expressed by APCs such as DCs and monocytes, lymphocytes only express P2XR.^48^ ATP sensing by the receptors P2×1R, P2×4R, P2×5R, and P2×7R activates effector T cells, which induces apoptosis in Tregs when ATP interacts with P2×7R^52 53^. Adenosine is recognized by two independent receptors: Adora2a (A2a) and Adora2b (A2b). The A2a receptor is constitutively expressed by T cells and has higher affinity for adenosine than the A2b receptor.^54^ When these receptors are stimulated with adenosine, cAMP is generated through adenylyl cyclase, which triggers protein kinase A and inhibits Tconvs proliferation and function.^55^ In murine tumor models, CD39 and CD73 expressed by Tregs reduce ATP and produce adenosine, which suppresses antitumor effector T cells. Tumor growth was suppressed in mice with either CD39^-/-^ or CD73^-/-^ Tregs compared with wild-type mice.^56 57^ When Tregs undergo apoptosis in the TME, apoptotic Tregs release a large amount of adenosine via ectonucleotidases, resulting in far stronger suppression of antitumor immunity.^58^

Through the multiple immunosuppressive mechanisms mentioned above, Tregs play a major role in resistance to PD-1 blockade therapy in many types of cancer. We have recently reported that PD-1^+^ Tregs could be activated by PD-1 blockade therapy with enhanced TCR and costimulatory signals, leading to PD-1^+^ Tregs with robust immunosuppressive function.^59^ Accordingly, the balance between PD-1^+^CD8^+^ T cells and PD-1^+^ eTregs is a novel biomarker for predicting the therapeutic effect of PD-1 blockade.^59^ Moreover, eTregs that express PD-1 could contribute to hyperprogressive disease after PD-1 blockade monotherapy in certain patients, particularly patients with liver metastases.^60^ Thus, patients with a high proportion of PD-1^+^ eTregs in the TME might need combination treatment that includes Treg-targeted therapy in addition to PD-1 blockade. Recently, liver metastases were shown to induce antigen-specific T cell suppression of systemic immunity against extrahepatic tumors in a preclinical model. Treg depletion reversed liver tumor-associated systemic immunosuppression, resulting in the accumulation of CD8^+^ T cells in extrahepatic tumors. Therefore, Tregs could contribute to systemic suppression of antitumor immunity in tumor-bearing hosts, particularly hosts with liver metastases.^61^ The detailed mechanisms underlying the systemic suppression of antitumor immunity by liver metastases are necessary to be elucidated.

## Mechanisms leading to Treg abundance in the TME

### Chemokine- and cytokine-dependent infiltration and conversion

**Figure 2:**
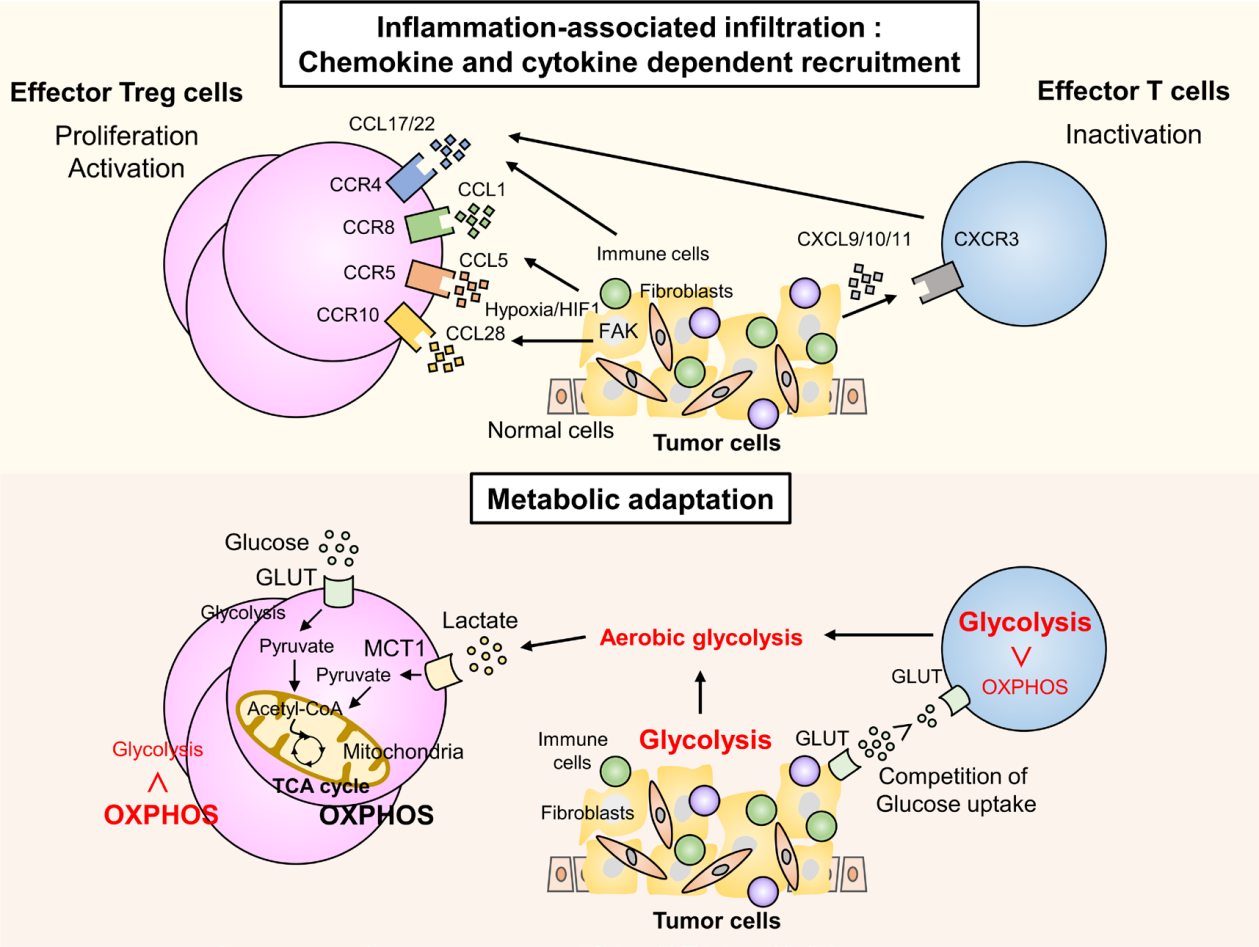
Mechanisms leading to Treg infiltration and adaptation in the inflamed TME. Inflammation-associated infiltration: chemokine and cytokine dependent recruitment. Tregs possess multiple chemokine receptors. Chemokine gradients such as CCR4-CCL17/22, CCR8-CCL1, CCR5-CCL5, and CCR10-CCL28 are involved in recruiting Tregs into the TME. Hyperactivation of focal adhesion kinase (FAK) is correlated with Treg infiltration and CD8^+^ T cell exclusion via regulation of the production of chemokines such as CCL5 by tumor cells. Tumor hypoxia induces the expression of CCL28 and promotes the recruitment of Tregs via CCR10. activated CD8^+^ T cells also produce CCL17/22 that recruit Tregs. On the other hand, chemokine gradients such as CXCR3-CXCL9/10/11 are involved in CD8^+^ T cell recruitment. Metabolic adaptation. Effector T cells and Tregs employ different metabolic system in normal versus inflammatory conditions. TCR stimulation provokes a specific metabolic program through the PI3K-mTOR signaling pathway, leading to increased uptake of glucose through glucose transporter (GLUT) to enhance aerobic glycolysis. Activated effector T cells shift their metabolic program from oxidative phosphorylation (OXPHOS) to aerobic glycolysis. Metabolic reprogramming in tumor cells changes the TME into a nutrient-restricted, lactate-rich, and hypoxic environment, which is unfavorable for the survival and function of effector T cells. FOXP3 plays an essential role in this distinct metabolic program of Tregs by suppressing glycolysis, promoting OXPHOS, and enabling the use of lactate through monocarboxylate transporter 1 (MCT1) as an energy source. While tumor-infiltrating non-Tregs convert pyruvate to lactate to maintain glycolysis, Tregs in the TME convert pyruvate to acetyl-CoA in the mitochondria to trigger the tricarboxylic acid (TCA) cycle, which provides a survival benefit to Tregs over effector T cells in the low-glucose, high-extracellular lactate TME. mTOR, mammalian target of rapamycin; PI3K phosphoinositide 3-kinase; TME, tumor microenvironment; Tregs, regulatory T cells.

Tregs have multiple chemokine receptors. Chemokine gradients such as CCR4-CCL17/22,^62^ CCR5-CCL5,^63^ CCR8-CCL1,^64^ and CCR10-CCL28^65^ can be involved in recruiting Tregs into the TME (figure 2). Tregs are generally recruited to sites of inflammation via the local cytokine milieu and are therefore detected with inflammatory cells, such as CD8^+^ T cells and myeloid cells in the inflamed tumors.^66^ In the inflamed tumors, inflammatory cells produce Treg-recruiting chemokines such as CCL22.^67 68^ CCR4, which is highly expressed by activated Tregs, has been implicated in their trafficking to nonlymphoid organs and tumors. For instance, CCR4-dependent and CCR5-dependent Treg infiltration is reportedly involved in breast cancer and lymphoma^69–71^ and pancreatic and squamous cell carcinoma,^63 72^ respectively. CCR4 is also needed for lymph node egress of activated Tregs that infiltrate the nascent TME in mouse melanoma with BRAF^V600E^.^73^ CCR8^+^ Tregs are recruited by CCR8 ligands, such as CCL1 and CCL18,^74^ to inflammatory sites like the TME. CCL1 not only recruits CCR8^+^ Tregs to tumors but also induces STAT3-dependent upregulation of FOXP3, CD39, and IL-10, which are crucial for Treg suppression, resulting in enhanced immunosuppressive activity in Tregs.^75^ Among a series of cytokine and chemokine receptors, CCR8 was most notably upregulated only in tumor-infiltrating Tregs in human breast cancer compared with normal tissue-resident Tregs,^76^ indicating that CCR8 is a promising therapeutic target for Tregs in the TME without eliciting systemic autoimmunity. Tumor hypoxia induces the expression of CCL28 and promotes the recruitment of Tregs through CCR10 in ovarian cancer. Tumor-infiltrating CCR10^+^ Tregs also produce vascular endothelial growth factor A (VEGFA) and establish a VEGFA-rich TME,^65^ which further promotes an immunosuppressive TME via VEGFA recruitment of Tregs.^77^

**Figure 3:**
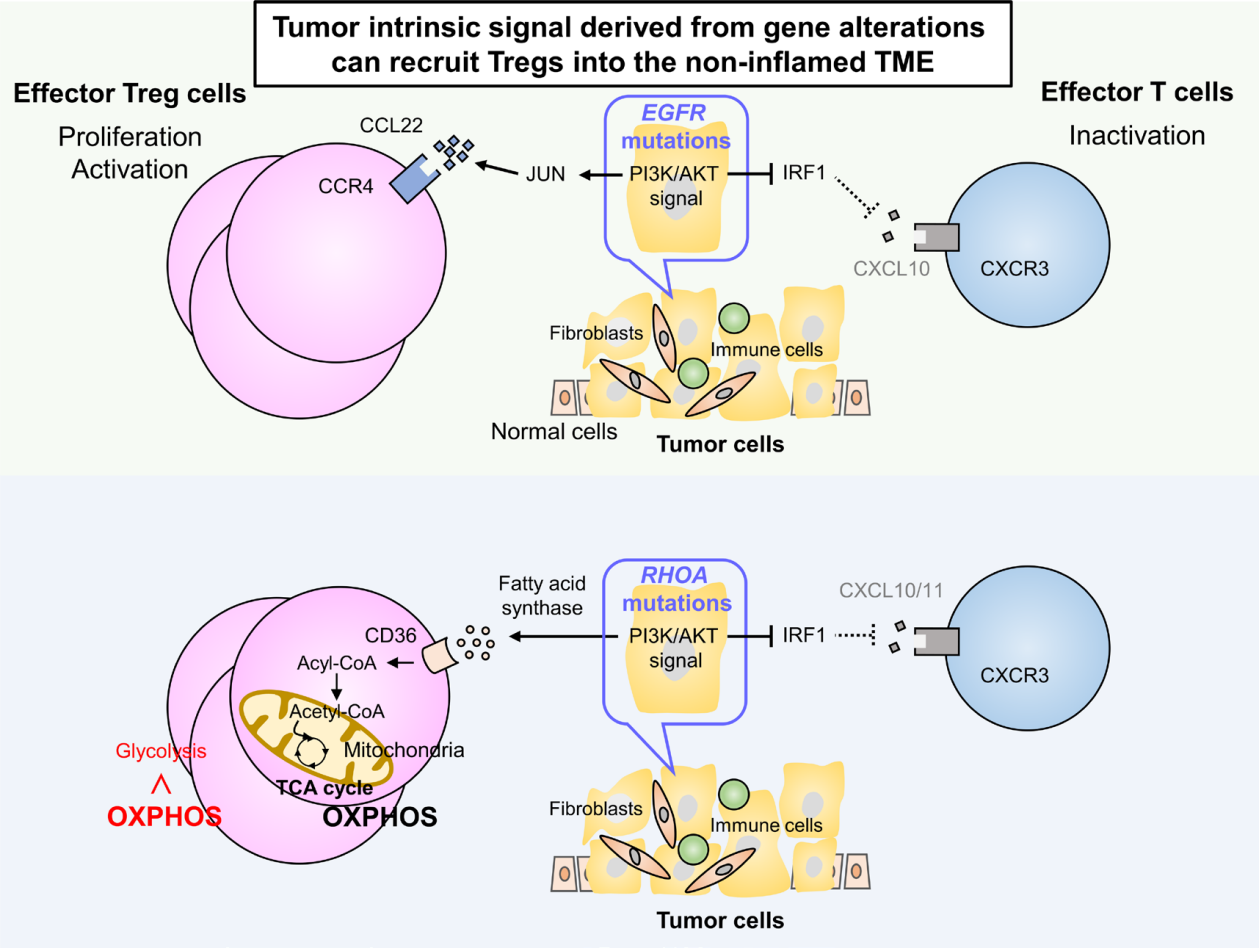
Mechanisms leading to Treg infiltration and adaptation in the non-inflamed TME. Tumor cell intrinsic signal-dependent infiltration. Certain gene alterations can modify the chemokine profile in tumor cells by modulating downstream signaling pathways. *EGFR* mutations in lung adenocarcinoma reduce CXCL10 production through interferon regulatory factor 1 (IRF1) inhibition. CCL22 production is increased via *JUN* induction, leading to CCR4-dependent Treg infiltration in the TME. In gastric cancers with *RHOA* mutations, CXCL10/11 levels are reduced through IRF1 suppression. *RHOA* mutations in gastric cancer produce large amounts of fatty acids through upregulation of fatty acid synthase (FASN) compared with tumors without *RHOA* mutations, leading to more Tregs and fewer effector T cells in the TME. Fatty acids produced in the TME can be used by Tregs as an energy source. Tregs use mechanisms in the fatty acid metabolism, such as upregulating the fatty acid transporter CD36, to adapt to the fatty acid-rich TME. FAK, focal adhesion kinase; GLUT, glucose through glucose transporter; OXPHOS, oxidative phosphorylation; PI3K, phosphoinositide 3-kinase; TME, tumor microenvironment; Tregs, regulatory T cells.

While the inflamed tumors commonly contain Tregs, abundant Tregs are sometimes detected in a subset of non-inflamed tumors, suggesting that mechanisms other than inflammation-associated infiltration are involved in recruiting Treg to the TME (figure 3). We discovered certain gene alterations that could modify tumor cells to produce chemokines through modulating downstream signaling pathways. Gain-of-function *EGFR* mutations found in lung adenocarcinoma^78^ are generally associated with non-inflamed tumors, but abundant Tregs have been detected in these tumors without the presence of inflammatory cells. *EGFR* mutations decrease CXCL10 production through IFN regulatory factor 1 (IRF1) inhibition, which negatively affects CXCR3-dependent CD8^+^ T cell recruitment to the TME. Moreover, CCL22 production is increased via *JUN* induction in downstream signaling of EGFR, leading to CCR4-dependent Treg infiltration into the TME.^79^ This mechanism accounts for why large numbers of Tregs accumulate in non-inflamed tumors with *EGFR* mutations. A similar phenotype was also observed in gastric cancers with *RHOA* Y42 mutation, which is loss-of-function mutation that reduces CXCL10/11 levels through IRF1 suppression.^80^ In addition, hyperactivation of focal adhesion kinase is correlated with Treg infiltration and CD8^+^ T cell exclusion through regulating chemokine production, including CCL5 production, by tumor cells.^81 82^

The specific cytokine and growth factor profile of the TME could induce the conversion of Tconvs to Tregs, which may contribute to the infiltration of Tregs into the TME. Previous studies have reported that immunosuppressive factors in the TME such as TGF-β can promote Tconvs to differentiate into pTregs with TCR stimulation and the CNS1 enhancer,^83^ which facilitates TGF-β-dependent FOXP3 induction in an animal model.^84^ Indoleamine 2,3-dioxygenase (IDO)-expressing myeloid cells might also promote the conversion of Tconvs to Tregs through an aryl hydrocarbon receptor (AHR).^85^ However, recent studies with human tumor samples have revealed that the overlap in TCR repertoires between intratumoral Tregs and Tconvs is limited,^21 76^ suggesting that Treg conversion is not a major source of tumor-infiltrating Tregs in humans. By contrast, tumor-resident Tregs react to mutated neoantigens and some immunogenic self-antigens in tumor cells,^21^ suggesting that thymus-derived tTregs undergo activation and clonal expansion in the TME in humans.

## Survival advantage of Tregs based on metabolic adaptations in the TME

Effector T cells and Tregs employ different metabolic systems in normal vs inflammatory conditions, which has been increasingly highlighted (figures 2 and 3).^80 86–88^ TCR stimulation provokes a specific metabolic program through the phosphoinositide 3-kinase (PI3K)-mammalian target of rapamycin (mTOR) signaling pathway, leading to increased uptake of amino acids and glucose to enhance aerobic glycolysis. Activated effector T cells shift their metabolic program from oxidative phosphorylation (OXPHOS) to aerobic glycolysis, which is necessary for their survival and function, resulting in a competition for glucose between effector T cells and tumor cells in the TME.^89^ Metabolic reprogramming in tumor cells changes the TME into a nutrient-restricted, lactate-rich, and hypoxic environment, which is unfavorable for the survival and function of effector T cells.^90^ However, Tregs can survive and retain their immunosuppressive function in such harsh conditions in the TME. FOXP3 plays an essential role in this distinct metabolic program of Tregs by suppressing glycolysis and promoting OXPHOS and nicotinamide adenine dinucleotide (NAD^+^) oxidation. As a result, Tregs can use lactate as an energy source.^91 92^ While tumor-infiltrating non-Tregs convert pyruvate to lactate to produce NAD^+^ to maintain glycolysis, Tregs in the TME convert pyruvate to acetyl-CoA in the mitochondria to trigger the tricarboxylic acid cycle, which provides a survival benefit to Tregs over other T cells, including effector T cells, in the low-glucose, high-extracellular lactate TME.^91^

In addition to lactate, fatty acids produced by tumor cells and stromal cells in the TME can be utilized by Tregs as an energy source for their survival and immunosuppressive function. Tregs use mechanisms in fatty acid metabolism, such as upregulating the fatty acid transporter CD36^93 94^ and sterol-regulatory-element-binding protein signaling,^95^ to adapt to the fatty acid-rich TME. The oxidative metabolism of lipids in Tregs decreases their demand for glucose and leads to resistance to fatty acid-induced cellular toxicity.^96^ Accordingly, even in low-glucose conditions, Tregs use fatty acids for their proliferation and immunosuppressive function.^80 94^ We have recently reported that *RHOA* Y42 mutation in gastric cancer produces large amounts of fatty acids through upregulation of fatty acid synthase (FASN) compared with tumors without *RHOA* Y42 mutation, leading to more Tregs and fewer tumor-infiltrating CD8^+^ T cells in the TME, which contribute to impaired sensitivity to PD-1 blockade therapy.^80^

When nTregs are stimulated with TLR ligands in mice, glycolysis is increased through mTOR complex 1 (mTORC1) signaling, which promotes proliferation but reduces the immunosuppressive function of Tregs.^86^ In addition, migratory capacity is dependent on the upregulation of glycolysis through mTORC2,^87^ suggesting that Tregs potentially use glycolysis for proliferation and migration. On the other hand, the immunosuppressive function of Tregs was diminished in mice harboring Treg-specific depletion of mitochondrial complex III, which impairs OXPHOS. However, proliferation and survival were not affected in these mice.^88^ A similar phenotype was observed in mice with Treg-specific depletion of the metabolic sensor liver kinase B1, which results in lethal autoimmunity due to the disruption of mitochondrial metabolism.^97 98^ Therefore, the immunosuppressive function of Tregs mainly depends on mitochondrial respiration and OXPHOS, which are regulated by FOXP3. The ability to use substrates from glycolysis or fatty acid oxidation provides Tregs with a functional and survival advantage in the TME. A direct relationship between glycolysis in tumor cells and intratumoral Treg stability has been demonstrated; glycolysis-low or defective tumors induce glycolysis in Tregs, resulting in functional destabilization of Tregs after CTLA-4 blockade therapy.^99^ This metabolic switch in Tregs is also found in a hypoxic environment. Hypoxia induces hypoxia-inducible factor (HIF)-1α expression, which stimulates glycolysis in Tregs through the upregulation of the glucose transporter and glycolytic enzymes and suppression of mitochondrial respiration.^93^ HIF-1α-deficient Tregs have impaired migration but increased immunosuppressive function in mice with brain tumors.^93^

IDO expression induces the catabolism of tryptophan and synthesis of kynurenine, which suppresses antitumor immunity in the TME. The level of IDO expression in the TME is strongly correlated with an increased number of intratumoral Tregs.^100^ Kynurenine interacts with the ligand-activated transcription factor AHR, which increases the proliferation and immunosuppressive activity of Tregs.^101 102^

## Treatment-Related Treg accumulation as an adaptive immune resistance mechanism in the TME

As described in previous sections, several mechanisms, including aberrant signaling in tumor cells based on gene alterations, can be involved in Treg recruitment into the TME before treatment (treatment-naive TME). On the other hand, treatments such as radiation therapy^103^ and CTLA-4 blocking antibodies^104^ promote the accumulation of Tregs in tumors, which is considered an adaptive immune resistance mechanism. Radiation therapy provides some favorable impacts on antitumor immunity through increasing antigen presentation and recruitment of cytotoxic immune cells, which is partly caused by DNA damage sensing through stimulator of interferon genes-dependent signaling.^105^ Tregs reportedly persist in the TME even after radiation therapy due to lower sensitivity to radiation than other lymphocytes,^106^ potentially leading to the development of an immunosuppressive TME in some types of cancer.^107 108^ Moreover, Treg depletion improved the sensitivity of tumors to radiation and inhibited metastasis in a preclinical model.^109^ Anti-CTLA-4 antibodies efficiently deplete Tregs through antibody-dependent cellular cytotoxicity (ADCC) in murine models,^110^ whereas the mechanism of action in patients remains controversial. Immunohistochemical analyses revealed that the number of Tregs increased after anti-CTLA-4 antibody treatment based on comparison of pre-treatment and post-treatment biopsy samples.^111^ Therefore, certain therapeutic interventions potentially enhance Tregs infiltration, which can impair the therapeutic outcome of subsequent treatments.

## Treatment strategies for highly specific Treg-targeted therapies

Given that Treg abundance in the TME is dependent on distinct mechanisms in each patient, particularly inflamed and non-inflamed tumors, we need to consider the immunological profile of the TME in each patient. Importantly, an initial study of Treg-targeted treatment clearly demonstrated that Treg depletion induces tumor regression in some tumor cell lines, such as Meth A and RL-male 1 (BALB/c radiation leukemia), but not in others such as AKSL2 and RL-female 8,^112^ indicating the importance of biomarkers for stratifying patients by the role that Treg suppression plays in tumor progression and survival when optimizing the clinical application of Treg-targeted therapy as immune precision medicine. Furthermore, when selecting the optimal treatment, the role of Tregs in the inflamed tumors or in non-inflamed tumors needs to be considered. Treg-targeted therapies discussed below (table 1) may be useful for both inflamed tumors and non-inflamed tumors. In particular, Tregs in non-inflamed tumors could be targeted with molecular-targeted therapy, given that they are generally recruited by specific mechanisms based on gene alterations, including *EGFR* and *RHOA* mutations in tumor cells.

**Table 1:** Therapies that target Tregs approved by the FDA or being evaluated in clinical trials

Name	Target	Clinical trial for solid tumors*
Targeting immunosuppressive mechanisms		
Ipilimumab	CTLA-4	FDA approved
Denileukin diftitox	CD25 (toxin conjugated)	FDA approved
ADCT-301	CD25 (ADC conjugated)	NCT03621982
RO7296682	CD25 (without IL-2 signal blockade)	NCT04158583, NCT04642365
TTX-030, SRF617	Ectonucleotidase CD39	NCT03884556, NCT04336098
LY3475070, Sym024, CPI-006, MEDI9447	Ectonucleotidase CD73	NCT04148937, NCT04672434, NCT03454451, NCT02503774
PBF-509, ciforadenant, NIR178	Adenosine receptor A2A	NCT02403193, NCT02655822, NCT03207867
Kinase inhibitors, ramucirumab	VEGFR2	FDA approved
IOA-244, AZD8186	PI3Kδ	NCT04328844, NCT04001569
Targeting chemokine receptors and immune checkpoints		
Mogamulizumab (KW-0761)	CCR4	FDA approved
FLX475	CCR4	NCT03674567
MEDI6469, PF-04518600, BMS 986178	OX40	NCT01862900, NCT02315066, NCT03831295
TRX518, BMS-986156, MEDI1873	GITR	NCT01239134, NCT04021043, NCT02583165
JTX-2011, KY1044, GSK3359609	ICOS	NCT02904226, NCT03829501, NCT02723955
Targeting metabolic adaptation		
VT1021	CD36	NCT03364400
AZD3965	MCT1	NCT01791595
Epacadostat, NLG802, GDC-0919	IDO	NCT01685255, NCT03164603, NCT02048709

*Representative ongoing clinical trials that include both monotherapy and combination therapy. Includes clinical trials that are completed or recruiting participants.

ADC, antibody-drug conjugate; CTLA4, cytotoxic T lymphocyte antigen 4; FDA, Food and Drug Administration; GITR, glucocorticoid-induced TNF receptor; IDO, indoleamine 2,3-dioxygenase; IL-2, interleukin 2; MCT1, monocarboxylate transporter 1; PI3K, phosphoinositide 3-kinase; Tregs, regulatory T cells; VEGFR2, vascular endothelial growth factor receptor 2.

## Targeting immunosuppressive mechanisms by Tregs

Anti-CTLA-4 monoclonal antibodies (mAbs) such as ipilimumab improve the immunological signature of the TME via Treg targeting. However, the detailed mechanism of action in clinical settings remains controversial. In mouse models, the antitumor immune responses induced by anti-CTLA-4 mAbs totally depend on the depletion of Tregs through Fc-mediated ADCC.^110^ In humans, while Treg depletion was not mainly involved in antitumor efficacy by ipilimumab,^104 111^ potential contribution of Fc-mediated ADCC was shown.^113^ Bispecific antibodies that target two molecules highly expressed by intratumoral Tregs, CTLA-4 and OX-40 or CTLA-4 and glucocorticoid-induced TNF receptor (GITR), had therapeutic effects in preclinical models.^114 115^ They might improve antitumor efficacy by efficiently depleting Tregs in humans. Targeting OX-40 and GITR will be discussed in the following section.

Since Tregs, especially intratumoral Tregs, express higher levels of CD25 than other effector T cells in human tumors, CD25 could be a crucial target for Treg depletion. Targeting CD25 expression by Tregs is therefore another option for modulating Treg function in the TME. Some therapeutic strategies targeting CD25 have been approved by the US Food and Drug Administration. For example, a fusion protein of IL-2 with diphtheria toxin, denileukin diftitox, is used in cutaneous T cell lymphoma. Although denileukin diftitox binds to CD25-expressing cells and kills them via the cytotoxic activity of diphtheria toxin, the efficacy for depleting Tregs was not sufficient in patients with melanoma.^116^ The novel anti-CD25 antibody RG6292 was developed to deplete Tregs selectively without disturbing IL-2 signaling in effector T cells^117^; it is under clinical evaluation (NCT04158583). Near-infrared photoimmunotherapy (NIR) targeting CD25 could also be a promising approach for local Treg depletion in tumors. NIR irradiation combined with an anti-CD25 antibody conjugated with a photoactivatable dye efficiently depleted Tregs in preclinical tumor models.^118^ In addition, ADCT-301, an antibody-drug conjugate against CD25, was developed for targeting CD25-expressing lymphomas.^119^ It might be used as another option to deplete Tregs in tumors; this approach is undergoing a clinical trial (NCT03621982).

Targeting adenosine production through the ectonucleotidases CD39 and CD73 expressed by Tregs can also be a promising target to augment antitumor immunity. Anti-CD39 and anti-CD73 antibodies that block ectonucleotidase activity, such as TTX-30, MEDI9447, and BMS-986179, are currently in clinical trials (NCT03884556, NCT03742102, and NCT02754141). In addition to inhibiting adenosine production, small molecule inhibitors of the adenosine receptor A2AR expressed by tumor-infiltrating T cells such as CPI-444, AZD4635, and PBF-509 prevent adenosine-dependent T cell suppression. Clinical trials for these inhibitors are underway (NCT02655822, NCT04089553, and NCT02403193).

VEGF receptor 2 (VEGFR2) is expressed by intratumoral Tregs, and VEGFA stimulation induced Treg proliferation in a preclinical model.^120^ We have shown that ramucirumab, an anti-VEGFR2 antibody, reduces the proliferation of eTregs in patients with gastric cancer.^121^ Thus, targeting the VEGFA-VEGFR2 axis might activate antitumor responses via reduction of Treg proliferation and infiltration in the TME.

Targeting FOXP3 to disrupt Tregs has also been attempted. AZD8701, an antisense oligonucleotide for FOXP3, partially reduced the expression of FOXP3 and its downstream transcriptional molecules in an in vitro experiment and humanized mouse models (https://cancerres.aacrjournals.org/content/79/13_Supplement/2713). A clinical trial of AZD8701 is underway (NCT04504669).

Another option is targeting the distinct intrinsic signal dependency between Tregs and Tconvs, such as TCR signaling.^122^ The tyrosine kinase inhibitor imatinib inhibits the oncogenic breakpoint cluster region-abelson (BCR-ABL) protein and also has various off-targets including lymphocyte-specific protein tyrosine kinase (LCK), which plays an important role in TCR signaling. An analysis of patients with chronic myelogenous leukemia treated with imatinib uncovered selective depletion of eTregs. A therapeutic concentration of imatinib specifically induced apoptosis in eTregs, but not in effector T cells such as CD8^+^ T cells. Since Treg survival is heavily dependent on continuous stimulation from the TCR signal,^123^ inhibition of LCK by imatinib selectively induced Treg apoptosis.^124^ The PI3K signaling pathway is also essential for T cell survival and function. While PI3Kδ isoform-specific PI3K inhibitor selectively depletes Tregs, the number of CD8^+^ T cells is increased through PI3Kδ specific inactivation in Tregs, resulting in the prevention of tumor progression and metastasis.^125 126^ In fact, since Tregs and effector T cells such as CD8^+^ T cells have differences in PI3Kδ dependency, the inhibitor could specifically target Tregs. Combination treatment consisting of the PI3Kδ inhibitor INCB050465 and the anti-PD-1 mAb pembrolizumab is currently being studied in a clinical trial (NCT02646748).

## Targeting chemokine receptors and immune checkpoint molecules on Tregs

Inhibiting chemokine-dependent migration of Tregs into the TME can sensitize tumors to immunotherapies (table 1). The anti-CCR4 antibody mogamulizumab reduces the number of CCR4^+^ Tregs in patients with solid tumors.^127 128^ The depletion of Tregs was confirmed in patients treated with mogamulizumab plus an anti-PD-1 antibody (nivolumab)^129^ in a clinical trial, suggesting that this combination is a promising option in combination cancer immunotherapies. A small-molecule antagonist of CCR4, FLX475, is currently under evaluation in a phase I/II study as monotherapy and in combination with pembrolizumab in advanced cancers (https://ascopubs.org/doi/abs/10.1200/JCO.2020.38.15_suppl.TPS3163)(NCT03674567). Blocking the CCL1-CCR8 axis is another option for Treg depletion in the TME.^75^ Two recent preclinical studies demonstrated that anti-CCR8 antibodies with Fc-dependent ADCC activity selectively deplete tumor-infiltrating Tregs due to prominent CCR8 expression by the activated Tregs in the TME, leading to long-lasting antitumor immune responses and synergistic antitumor effects with PD-1 blockade.^130 131^

Immune checkpoint molecules that are highly expressed by Tregs, such as OX40, GITR, and ICOS, could also be therapeutic targets. Several studies have shown that the stimulation of these receptors reduces the immunosuppressive function of Tregs, leading to the activation of effector T cells.^132 133^ Antibodies that act as agonists of OX40, such as MEDI6469^134^ (NCT02274155), GITR, such as MK-4166^135^ (NCT02132754), and ICOS, such as KY1044^136^(NCT03829501), are currently being investigated.

## Targeting metabolic adaptation of Tregs to the TME

Tregs can use free fatty acids and lactate, which provides metabolic advantage over effector T cells especially in the harsh-nutrient TME. To disturb the metabolic adaptation of Tregs, the fatty acid transporter CD36 and the lactate transporter monocarboxylate transporter 1 (MCT1), which are required for fatty acid and lactate uptake, respectively, can be important targets for reducing the number of Tregs and hampering their function in the TME. Inhibition of CD36 or MCT1 reduced Treg abundance in the TME and improved the sensitivity of PD-1 blockade in preclinical models.^80 92 94^ In addition to targeting fatty acid uptake, blocking FASN with an acetyl-CoA carboxylase inhibitor (5-(tetradecyloxy)−2-furoic acid) and blocking fatty acid oxidation with a carnitine palmitoyltransferase 1a inhibitor, suppressed the proliferation and immunosuppressive function of Tregs.^93 137^ Elevated extracellular lactic acid levels reduce the function of effector T cells. Therefore, a lactate dehydrogenase A inhibitor can potentially rescue the effector function of T cells by reducing L-lactate production.^91 138^

Kynurenine produced with IDO activity on tryptophan, which interacts with AHR, increases the number of Tregs and tolerogenic myeloid cells in the TME. IDO-riched tumors have an activated AHR pathway, which is associated with resistance to PD-1 blockade.^101^ Targeting the IDO-Kynurenine-AHR axis could be a promising approach for improving the sensitivity of ICI by decreasing Tregs.

## Conclusions and future directions

The involvement of multiple mechanisms in Treg infiltration, activation, and survival in the TME has been revealed both in inflamed and non-inflamed tumors. The mix of mechanisms affecting Treg infiltration, activation, and survival in the TME might vary by patient. Gene alterations in tumor cells not only determine immunogenicity and inflammatory status but also contribute to the modulation of immune cell infiltration and survival in the TME, such as Treg infiltration based on chemokine profiles and Treg activation based on metabolic changes, which could be targeted by specific kinase inhibitors. Therefore, the mechanisms that mainly contribute to Treg abundance in the TME need to be characterized through both immunological and metabolic profiling based on gene alteration and targeted with immune precision therapy. Mechanism-based Treg-targeted therapy shows promise for improving current immunotherapies.
