# Correction: *Mechanisms of regulatory T cell infiltration in tumors: implications for innovative immune precision therapies*

**DOI:** 10.1136/jitc-2021-002591corr1

**Published:** 2021-09-13

Koyama S, Nishikawa H. Mechanisms of regulatory T cell infiltration in tumors: implications for innovative immune precision therapies. *J Immunother Cancer* 2021;9:e002591. doi: 10.1136/jitc-2021-002591

This paper has been updated to amend author order.

